# Applicability of devices available for the measurement of intracompartmental pressures: a cadaver study

**DOI:** 10.1186/s40634-022-00529-0

**Published:** 2022-09-27

**Authors:** Sanne Vogels, Ewan D. Ritchie, Djuna de Vries, Gert-Jan Kleinrensink, Michiel H. J. Verhofstad, Rigo Hoencamp

**Affiliations:** 1 Department of Surgery, Alrijne Hospital, Simon Smitweg 1, 2353 GA Leiderdorp, The Netherlands; 2 Trauma Research Unit, Department of Trauma Surgery, Erasmus Medical Center, Rotterdam, the Netherlands; 3 Department of Neuroscience, Erasmus Medical Center, Rotterdam, the Netherlands; 4 Defense Healthcare Organization, Ministry of Defense, Utrecht, the Netherlands; 5 Department of Surgery, Leiden University Medical Center, Leiden, the Netherlands

**Keywords:** Compartment pressure, Chronic Exertional compartment syndrome, Manometers, Needles, Compartment pressure monitor

## Abstract

**Purpose:**

The indication for surgical treatment of the chronic exertional compartment syndrome is evaluated by measuring intracompartmental pressures. The validity of these invasive intracompartmental pressure measurements are increasingly questioned in the absence of a standardized test protocol and uniform cut-off values. The aim of the current study was to test compartment pressure monitors and needles for uniformity, thereby supporting the physician’s choice in the selection of appropriate test materials.

**Methods:**

A compartment syndrome was simulated in embalmed above-knee cadaveric leg specimen. Four different terminal devices (Compass manometer, Stryker device, Meritrans transduce, and arterial line) were tested with 22 different needle types. Legs were pressurized after introduction of the four terminal devices in the anterior compartment, using the same needle type. Pressure was recorded at a 30-second interval for 11 minutes in total. Before and after pressurization, the intravenous bag of saline was weighed.

**Results:**

The simulation of a compartment syndrome resulted in intracompartmental pressure values exceeding 100 mmHg in 17 of the 22 legs (77%). In the other five legs, a smaller built-up of pressure was seen, although maximum intracompartmental pressure was in between 70 and 100 mmHg. The intraclass correlation coefficient was above 0.700 for all possible needle types. Excellent to good resemblance was seen in 16 out of 22 instrumental setups (73%). The mean volume of saline infusion required in runs that exceeded 100 mmHg (309 ± 116 ml) was significantly lower compared to the legs in which 100 mmHg was not achieved (451 ± 148 ml; *p* = 0.04).

**Conclusion:**

The intracompartmental pressure recordings of the four terminal devices were comparable, when tested with a standardized pressurization model in a human cadaver model. None of the included terminal devices or needle types were found to be superior. The results provide evidence for more diverse material selection when logistic choices for intracompartmental pressure measurement devices are warranted.

**Level of evidence:**

Level IV.

**Supplementary Information:**

The online version contains supplementary material available at 10.1186/s40634-022-00529-0.

## Introduction

The Chronic Exertional Compartment Syndrome (CECS), a disorder mostly affecting the lower extremities, can result in significant morbidity and limitations in activities of athletes, military personnel and recreational sportsmen [12]. It is characterized by a sensation of tightness and pain during or after performing repetitive physical activity. The etiology of CECS is largely unknown but may be related to pathologically elevated intracompartmental pressures (ICP) following muscular expansion during exercise [4, 5, 27, 30]. In the diagnostic work-up, these ICPs are measured using an invasive needle or catheter manometry before, during, or after provocative exercise [23]. However, consensus regarding the cut-off value indicative for CECS is currently lacking [1, 17].

Invasive ICP manometry is considered a user-dependent assessment, since a reproducible or standardized protocol is absent and readings were shown to be dependent on education [8, 11]. Hislop and Tierney [8] concluded that discrepancies were mainly found in the number of legs or compartments that were tested, the exercise used to provoke symptoms, and the position of the leg during needle placement or pressure recording. Also, physicians often do not use ultrasound to confirm correct placement of the needle or catheter, even though studies denounce a clear risk for inaccurate placement, particularly in the deep compartment [28, 29].

In addition, the setup used to perform ICP measurements is not standardized and can be performed with various types of available manometers, needles, and catheters. Several instrumental setups appear to be acceptable to perform ICP measurements, under the condition that a locally established protocol with cut-off values is adhered to [17]. However, studies demonstrated that absolute ICP values vary when either a slit catheter, side-port needle, or straight needle was used in the same experimental setup [2, 3, 7, 14, 22]. Boody et al. [2] declared to have a preference for the handheld Stryker manometer over the Whitesides [25], whereas Hammerberg et al. [7] compared a standard electronic arterial pressure monitor (Datex-Ohmeda S/5) with the Whitesides technique and did not find a significant difference between the two pressure monitors at all. Although all setups are based on the principle of installing a continuous saline fluid column between tissue in a compartment and the pressure device, exact clinimetric properties for these instrumental setups is often missing.

In the absence of a standardized test protocol and uniform cut-off values, the validity of ICP manometry is increasingly questioned [1, 8, 11, 17]. In addition, with the commonly applied Stryker device being no longer commercially available in Europe, physicians are forced to look for alternatives. As different setups (i.e. combination of different devices and needles) might result in different outcomes, information concerning the theoretically most precise setup might be helpful to support clinicians in their choice for alternative setups. Therefore, the aim of this study was to contribute to (partial) standardization of the diagnostic test protocol for CECS and to support the physician’s choice in the selection of the appropriate ICP measurement device and needle combination. This study further evaluated the results obtained during a comparable experiment conducted with a simulation model in the laboratory and was hypothesized to find comparable results [24].

## Materials and methods

A cadaver study was performed. Under [Dutch] law, and under these conditions no approval of the medical research ethics committee was required. The study was performed in the Procedural Skills Laboratory of an academic hospital ([Erasmus MC, Rotterdam, the Netherlands]). Post-mortem human specimen (PMHS) were obtained through the Department of Anatomy, which were flushed with Anubifix® to regain elasticity after rigor mortis and embalmed with a 4.4% formalin solution. All total body donors were part of the national donor program and have given written consent for tissue donation for educational and scientific purposes before passing away. Due to [European] privacy regulations, medical history was only available to the general practitioner of the donor and not to the receiving academic medical center.

### Equipment

**Fig. 1:**
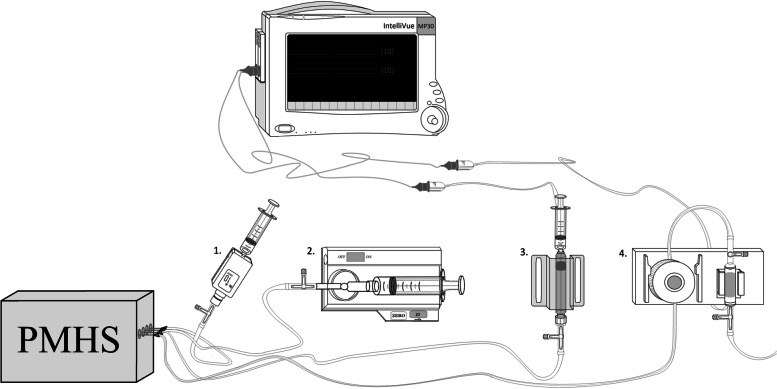
Schematic representation of the experimental setup. Four different terminal devices were evaluated using the simulation of a compartment syndrome in a post-mortem human specimen (PMHS): 1. Compass UniversalHg; Iskus Health, Ltd., Dublin, Ireland, 2. Stryker Intracompartmental Pressure Monitor System; Stryker, Kalamazoo, Michigan, 3. Meritrans DTXPlus® Disposable Transducers; Merit Medical Systems, Jordan, Utah & IntelliVue MX500 monitor; Philips, Eindhoven, the Netherlands, 4. Xtrans® system; CODAN, Lensahn, Germany & IntelliVue MX500 monitor; Philips, Eindhoven, the Netherlands

Four manometer devices were tested: (1) A commonly used arterial line (Xtrans® system; CODAN, Lensahn, Germany & IntelliVue MX500 monitor; Philips, Eindhoven, the Netherlands); (2) the Stryker device (Stryker Intracompartmental Pressure Monitor System; Stryker, Kalamazoo, Michigan); (3) the Compass pressure monitor (Compass UniversalHg; Iskus Health, Ltd., Dublin, Ireland); and (4) the Meritrans transducer (Meritrans DTXPlus® Disposable Transducers; Merit Medical Systems, Jordan, Utah & IntelliVue MX500 monitor; Philips, Eindhoven, the Netherlands). The four terminal devices were each combined with 22 different commercially available needles (Supplementary Table 1), all varying in diameter and length, resulting in a total of 88 instrumental variations (Table 1). Connection between the continuous water column, free of air bubbles, and one of the four terminal devices was established using saline filled intravenous tubing with a three-way stopcock. A schematic representation of this setup can be found in Fig. 1.

**Table 1:** Material availability

All materials and resources were arranged in the absence of interference by parties other than the appointed research funding. None of the mentioned commercial organizations were approached to participate actively, nor donated goods or materials.

### Experimental compartment model

Above-knee amputated legs of PMHS were used to create a compartment syndrome model, comparable to previously published studies [11, 14, 15, 18–20]. The lower limb was visibly free of trauma or postsurgical changes. A 14-gauge angiocatheter was inserted in the proximal part of the anterior compartment of the lower leg and connected to the intravenous bag using standard IV tubes and connectors. Normal 0.9% saline (B. Braun Healthcare Corporation, Oss, the Netherlands) was infused using a manual pressure infusion bag with manometer.

### Experimental procedure

All measurements were performed on two consecutive days, to keep location, room temperature, and humidity at a constant. The experimental setup was left unchanged in a secured room throughout this period.

#### Development of a decay model

A decay model, inspired by Teng et al. [20], was developed and tested with three embalmed PMHS legs to optimize pressurization and to evaluate natural pressure decay over time. In addition, a fresh-frozen PMHS leg was tested with the decay model to confirm the previously described similarities between fresh-frozen and embalmed PMHS types [6, 9, 21] and to justify the selection of embalmed PMHS for the current experiment.

**Fig. 2:**
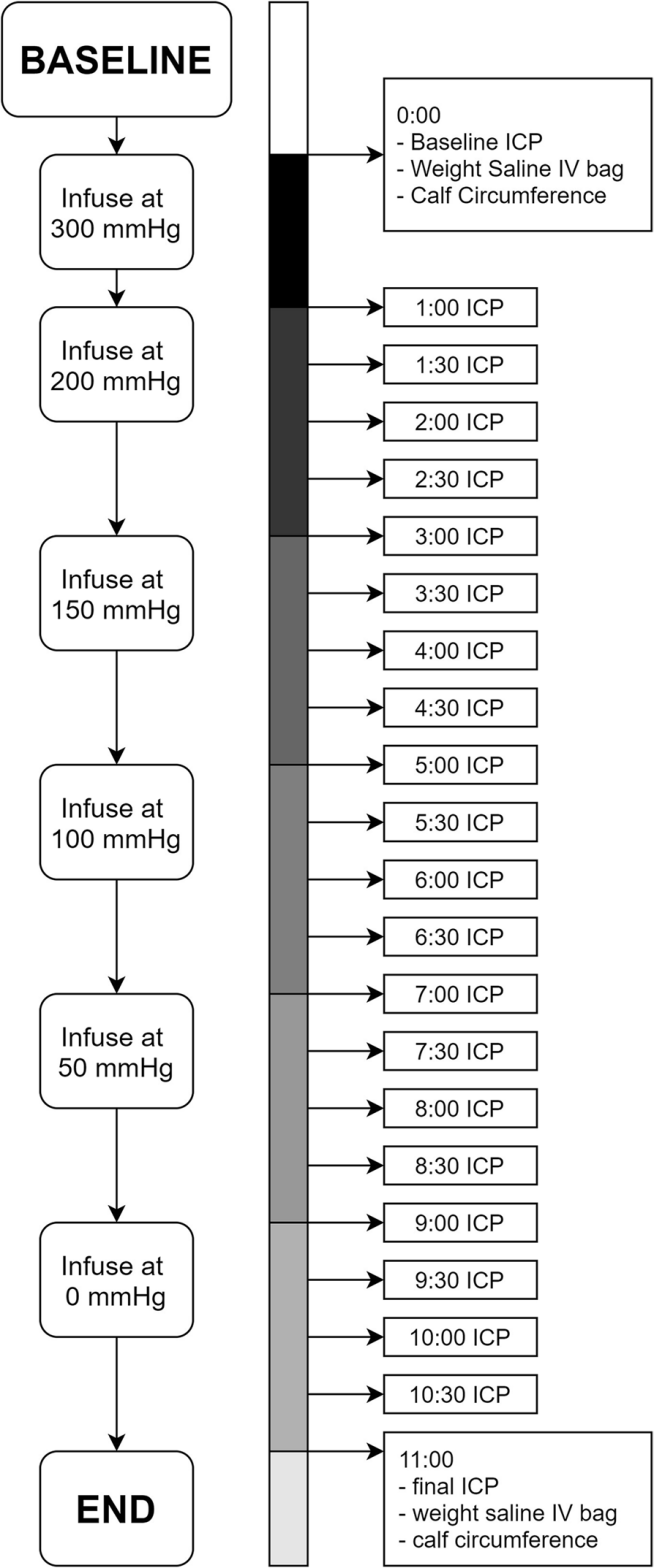
Timeline of the experiment

The most commonly encountered instrumental setup (Side-port needle & Stryker Intracompartmental Pressure Monitor; Stryker Instruments, Kalamazoo, Michigan) was used to measure ICP continuously proximal, central, and distal in the anterior compartment. In the first minute, saline was infused with a pressure of 300 mmHg (Fig. 2). Subsequently, pressure was lowered to 200 mmHg, allowing for equilibration of the saline infusion. After every 2 min, 50 mmHg was released from the pressure bag. ICP was recorded at baseline and hereinafter with a 30-second interval. The saline fluid bag was kept at a constant height of 75 cm above the PMHS leg at all times. One hour hereinafter, the same run was repeated with the exact same experimental setup, to see whether a leg could be used repeatedly.

#### Pressurization

**Fig. 3:**
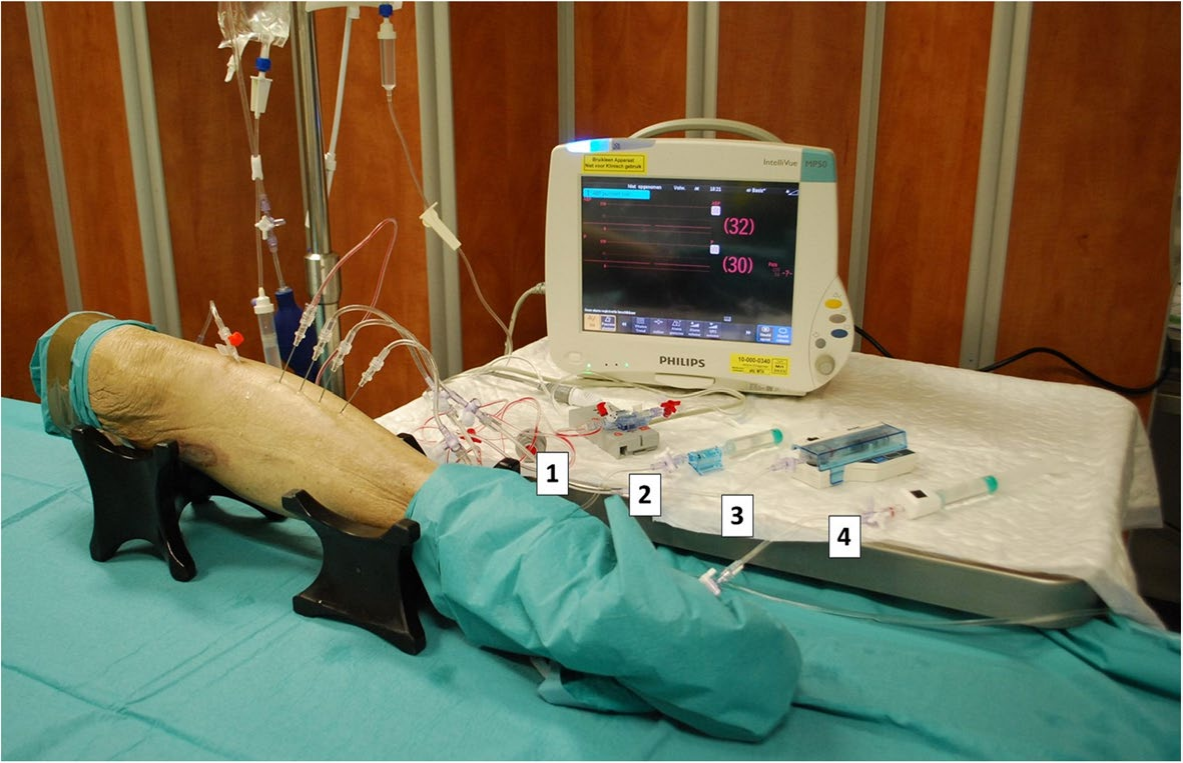
Example of a limb with the experimental setup installed, using the Stryker side-ported needle. The four different terminal devices are: 1. Xtrans® system; CODAN, Lensahn, Germany & IntelliVue MX500 monitor; Philips, Eindhoven, the Netherlands, 2. Meritrans DTXPlus® Disposable Transducers; Merit Medical Systems, Jordan, Utah & IntelliVue MX500 monitor; Philips, Eindhoven, the Netherlands, 3. Stryker Intracompartmental Pressure Monitor System; Stryker, Kalamazoo, Michigan, 4. Compass UniversalHg; Iskus Health, Ltd., Dublin, Ireland

Prior to the start of the experimental procedures, all terminal devices were calibrated as prescribed. Every type of needle was tested on a different leg, meaning 22 specimens were required to test all available needles. Per leg, four identical needles were introduced at a right angle to the surface, halfway of the anterior compartment (Fig. 3). To these needles, the four different terminal devices were connected with saline filled IV tubing. All instrumental setups were flushed with 0.5–1 ml saline and pressure was allowed to equalize so baseline pressure could be recorded. Pressurization was executed according to the previously defined decay model with continuous pressure monitoring by all four terminal devices. Pressure was recorded at a 30-second interval for 11 minutes in total (Fig. 2).

#### Additional data collection

Before and after pressurization, the intravenous bag of saline was weighed (to determine the amount of saline infused) and calf circumference of the leg was measured. Also, the leg was monitored at all times for possible signs of leakage (e.g. edema formation in the ankle, saline leakage from the amputation site, or absence of bulging of the anterior compartment).

### Statistical analysis

Statistical analysis was performed using SPSS statistics (v25, IBM Corporation, Armonk, New York). All recordings per needle were plotted in one graph. Subsequently, the Intraclass Correlation Coefficient (ICC) [10] was used to compare the recordings of the four different terminal devices, thereby evaluating potential presence of instrumental bias. This was done using a two-way mixed method with absolute measures. Reliability and precision were quantified using the 95% confidence interval (CI) (poor < 0.500, moderate 0.500–0.750, good 0.750–0.900, or excellent > 0.900).

Additionally, the correlation between the amount of saline infusion and the overall change in ICP values was analyzed for instrumental setups with an ICC > 0.700. For this analysis, the 22 trials were arranged in two groups, with the first group comprising all trials in which ICPs exceeded 100 mmHg and the second group those in which this elevation could not be achieved. The amount of saline infusion in milliliters was calculated by subtracting the weight of the saline IV bag before and after the decay model, followed by a conversion with standard values for the density of saline solution at a given temperature. Furthermore, the area under the curve was calculated for the four different terminal devices per needle type. The mean AUC per needle type was correlated to the amount of saline infusion using a Pearson Correlation. An independent sample T-test was used to compare the mean of saline infusion needed in measurements that did or did not exceed 100 mmHg. Two-sided *p*-values ≤0.05 were considered significant.

## Results

The 25 experimental procedures (3 test procedures + 22 procedures with possible needle and device combinations) and corresponding ratings were independently performed by the three assigned researchers ([SV, ER and DdV]) according to the described decay model. The four devices were similar in ease of use and no technical difficulties were encountered. Three embalmed and one fresh-frozen legs used to optimize the decay model showed that elevated pressures up to 204 mmHg could be simulated with this protocol. Moreover, these runs revealed that 2 minutes of equilibration was sufficient for the ICP to stabilize and that the decay model could run comparably in both the embalmed and the fresh-frozen leg. Also, similar pressures were recorded irrespective of needle location (i.e. proximal or distal). A repeat run in the same leg did not result in comparable or stable elevated pressures.

### Pressurization with the decay model

**Fig. 4:**
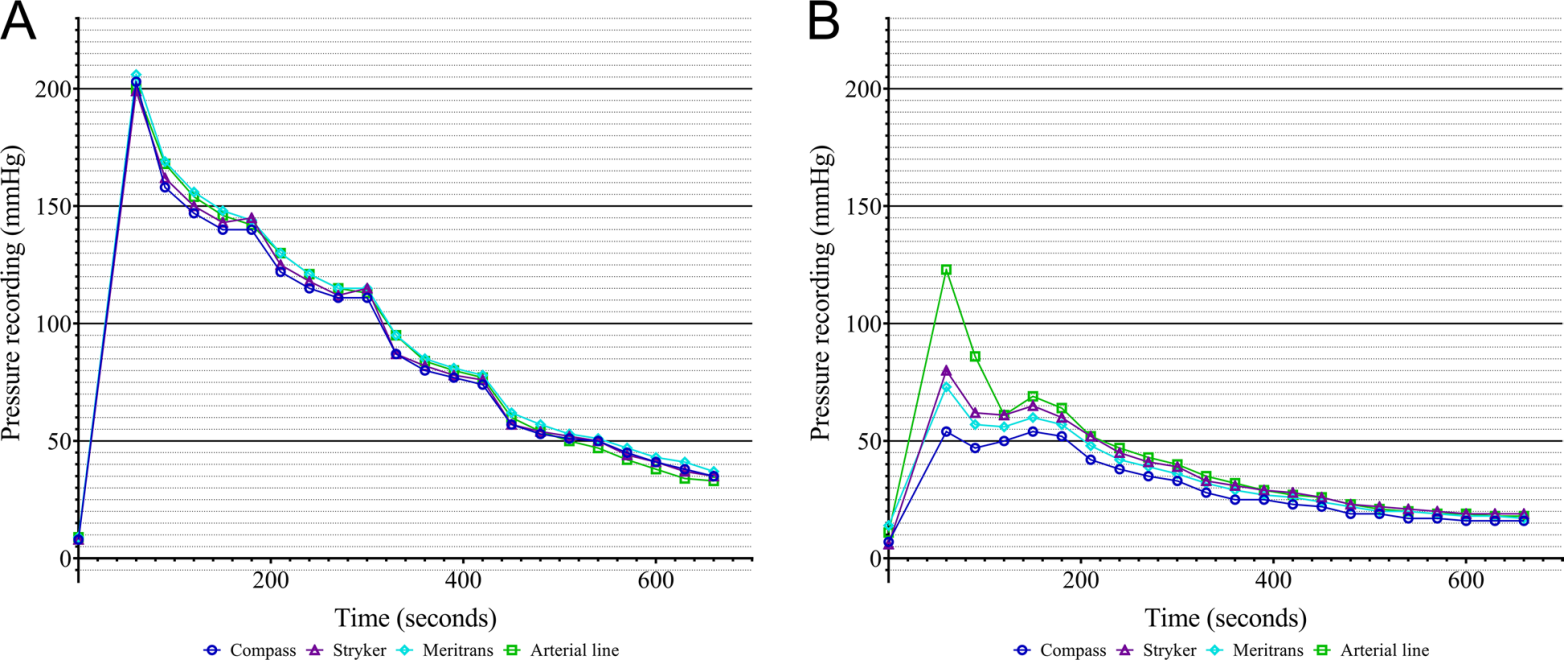
Graphical illustration of the pressure recordings throughout the decay model for the four terminal devices (Compass manometer, the Stryker device, Meritrans transducer, and an arterial line) in combination with (**A**) a Venflon Pro Safety catheter (18 gauge) and (**B**) a Microlance straight needle (21 gauge)

The compartment syndrome was simulated successfully in all 22 legs. At baseline, a mean ICP of 9 (±4) mmHg with a range of 3 to 24 mmHg was recorded. With this decay model, ICP values exceeding 100 mmHg could be provoked in 17 of the 22 legs (77%; Table 2). In the other five legs, a smaller built-up of pressure was seen, although maximum pressures exceeded 70 mmHg. However, no visible signs of saline leakage were witnessed. Examples of a successful and less successful buildup of 100 mmHg with the decay model are depicted in Fig. 4A and B, respectively. Additional graphical illustrations of the recorded pressures for the four terminal devices (with the side-ported needle of Stryker, the C2Dx slit catheter, or the Sonoplex straight needle (21 gauge)) can be found in Supplementary Fig. 1 (A-C).

**Table 2.** Overview of change in calf circumference, the amount of saline infusion needed, the maximum pressurization, and the Intraclass Correlation Coefficient (ICC) with the respective confidence interval (CI) after running the decay model per needle type per post mortem human specimen

	**Δ calf circumference (centimeters)**	**Saline infusion (ml)**	**Maximum Pressurization**		ICC(95% Confidence Interval)*
			>100 mmHg	<100 mmHg	
**Catheters**					
Slit Catheter, C2Dx	1	222	X		0.834 (0.593-0.932)^c^
Venflon Safety Pro, 22 gauge, BD	1	401		X	0.968 (0.898-0.988)^b^
Venflon Safety Pro, 20 gauge, BD	4	378	X		0.996 (0.984-0.999)^a^
Venflon Safety Pro, 18 gauge, BD	1	347	X		0.996 (0.990-0.998)^a^
Venflon Safety Pro, 17 gauge, BD	2	420	X		0.893 (0.809-0.948)^b^
Venflon Safety Pro, 16 gauge, BD	1	302	X		0.990 (0.969-0.996)^a^
Venflon Safety Pro, 14 gauge, BD	4	308		X	0.863 (0.696-0.941)^c^
Intranule, 18 gauge, Vygon	2	358	X		0.994 (0.976-0.998)^a^
Intranule, 16 gauge, Vygon	1	448	X		0.896 (0.607-0.965)^c^
Intranule, 14 gauge, Vygon	3	647		X	0.992 (0.984-0.997)^a^
Intranule, 13 gauge, Vygon	2	564		X	0.948 (0.897-0.976)^b^
**Straight needles**					
Microlance needle, 25 gauge, BD	2	248	X		0.881 (0.790-0.943)^b^
Microlance needle, 23 gauge, BD	2	222	X		0.877 (0.765-0.943)^b^
Microlance needle, 21 gauge, BD	3	335		X	0.855 (0.717-0.934)^c^
Microlance needle, 18 gauge, BD	1	145	X		0.943 (0.732-0.982)^c^
Microlance needle, 16 gauge, BD	2	116	X		0.994 (0.977-0.998)^a^
Sonoplex facet tip, 22 gauge, Pajunk	3	347	X		0.838 (0.722-0.920)^c^
Sonoplex facet tip, 21 gauge, Pajunk	4	417	X		0.919 (0.840-0.963)^b^
Sonoplex facet tip, 20 gauge, Pajunk	0	234	X		0.978 (0.956-0.990)^a^
**Side-ported needles**					
Side-ported needle, C2Dx	3	526	X		0.989 (0.973-0.995)^a^
Sonoplex Sprotte tip, Pajunk	1	157	X		0.890 (0.777-0.950)^b^
Side-ported needle, Stryker	1	370	X		0.998 (0.993-0.999)^a^

* Excellent reliability with CI >0.900 is represented in ^a^, good reliability with CI 0.900-0.750 in ^b^, moderate reliability with CI 0.750-0.500 in ^c^, and poor reliability with CI < 0.500 in red

The ICC comparing the results of the four terminal devices per needle was above 0.700 for all possible combinations (Table 2). Excellent to good reliability was seen in 16 out of 22 instrumental setups (73%).

### Correlation saline infusion and pressurization

**Fig. 5:**
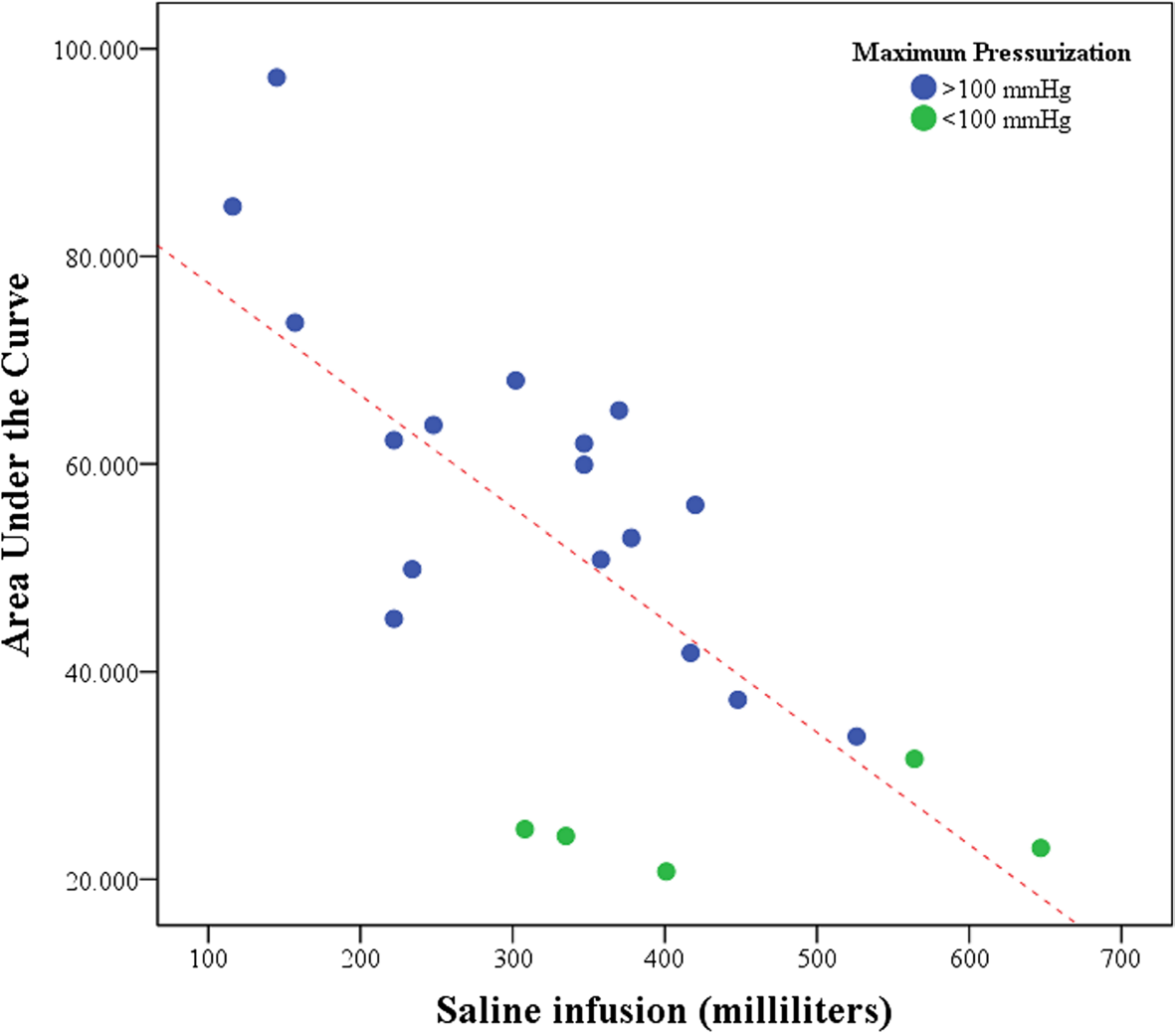
Correlation between the amount of saline infusion and the overall change in ICP values (Area Under the Curve). A statistically significant negative relation was found amongst all 22 trials (*r* = − 0.709, *p* < 0.01). Also, the mean volume of saline infusion required in runs that exceeded 100 mmHg was significantly lower compared to those which did not exceed this threshold (*p* = 0.04)

The mean volume of saline infused during the decay model in the 22 legs was 343 ± 133 ml. The amount of saline infused and the area under the pressurization curve were shown to have a statistically significant linear relationship (*r* = − 0.709, *p* < 0.01; Fig. 5), indicating that an increase in saline infusion resulted in a smaller overall pressurization with the decay model. Moreover, the mean volume of saline infusion required in runs that exceeded 100 mmHg (309 ± 116 ml) was significantly lower compared to those which did not exceed this threshold (451 ± 148 ml; *p* = 0.04).

## Discussion

The presented PMHS model evaluated four different ICP measurement devices and revealed that similar ICP pressures were recorded by all devices, irrespective of the type of needle used in the instrumental setup. All 22 possible device and needle combinations were shown to record comparable absolute measures. In nine of these combinations, the confidence interval denoted an excellent reliability and precision in measurements. No superiority for a specific device and needle combination was reported.

In daily clinical practice, the presence of CECS is often confirmed using ICP manometry. Up until recently, this was mainly done by using the Stryker Intracompartmental pressure Monitor System (Stryker, Kalamazoo, Michigan) as instrumental setup. However, with these materials recently becoming unavailable worldwide, the clinical practice of sports physicians and surgeons was challenged. Especially since the search for appropriate alternative setups was hampered by the fact that clinimetric properties of alternative setups are often missing. Subsequently, professionals were required to work with less familiar instrumental setups, in a time when validity of the ICP manometry is already increasingly debated due to the absence of a uniform cut-off value or test protocol.

The present study provides an updated overview regarding the reliability of available ICP measurement materials in clinical practice. In the past, studies reported that ICP measurements were preferably obtained using either a side-ported needle or a slit catheter, rather than a simple needle [2, 14]. Contrary to this, a more recent study reported not to find superiority amongst these needles and concluded all needles could be used with clinical confidence [7]. The present study confirms the data from the latter study. Looking at the terminal devices, the conventional arterial line manometer or the device by Stryker was shown to be superior to the Whitesides apparatus [2, 22, 25, 26]. If any of these materials would be unavailable, a single study indicated that an IV pump with pressure sensor would be a suitable and satisfactory alternative [22].

Naturally, products that came available hereinafter are not mentioned in previous literature. A first attempt in doing so involved an ex vivo experiment with a graduated water column, comparable to Uliasz et al. [22] and Boody et al. [2]. This study concluded that only the conventional arterial line and Stryker materials remained reliable in the measurement of ICP values in a porcine gluteal muscle sample [24]. The two other and newly developed terminal devices (Compass UniversalHg and the pressure transducer by Meritrans) seemed to lose accuracy after the addition of a muscle sample in the model.

The findings of this previously performed experiment with the water column model [24] are contradicted by this experiment with a PMHS model. The current results namely show that all terminal devices report comparable ICP values with an excellent to moderate reliability after introduction into muscle tissue, without superiority for a specific type of needle. This difference could have been instigated by the way the compartment syndrome was induced in the current PMHS model; with saline being infused directly and under high pressure in the compartment of an embalmed leg, rather than intravenously in a fresh frozen body [13]. Because of this, the saline could have collected in the intracompartmental space, without diffusion into the embalmed muscle tissue. It is therefore hypothesized that current measurements possibly reflect pressures of free-floating saline in the compartment, rather than actual tissue pressure of the tibialis anterior muscle. If that is the case, this PMHS model more closely resembles the graduated water column without a porcine muscle sample at the bottom. However, this explanation is challenged by the finding that a repeat run of the decay model gave overall higher and less variable pressure values, suggestive for the presence of diffused saline in the muscle tissue. So, although the presented findings might imply that all available materials are suitable for clinical ICP measurement, results have to be interpreted with caution and preferably tested with living patients first.

The variability in pressurization in the different legs might be due to a variation in the anatomy of the tested subjects. A sizable case series comparing three methods for ICP manometry (the Stryker materials, an arterial line, and an electronic transducer-tipped catheter) already suggested that the limited reliability of all single pressure measurements might be due to heterogeneous anatomy of the tested muscle compartments [3]. In the current study it was noted that throughout the experiment a wide range of ICP values could be achieved using different quantities of saline despite standardization of the pressurization model. This fluctuation in volume could be inherent to variation in the size of the muscle compartment, elasticity of the embalmed fascia or the amount of subcutaneous fat in a leg specimen. Also, although no visible signs of leakage were detected, the significant difference in saline infusion between legs that could or could not exceed 100 mmHg pressurization (Fig. 5) makes leakage via for example facial hernias highly suspected. Nevertheless, with all trials showing a minimum elevation of at least 70 mmHg, all simulations did exceed Pedowitz’ 30 mmHg threshold [16] and could therefore still be regarded as successfully.

The study was subject to several limitations. Possible generalization of the current study result is limited due to several reasons, the most prominent being the use of an ex vivo environment and the use of a different PMHS specimen per needle tested. Also, contrary to the experiments performed in a graduated water column, the actual ICP value inside the compartment throughout trial with the decay model was not known. Therefore, measurements in the current study could not be compared to a known reference pressure, but only to the pressure recordings of the other devices. So, the results reflect the extent to which the terminal devices agree, rather than knowing whether the devices actually measured the physiological ICP. Nevertheless, by standardizing the pressurization model and most of the environmental factors, this should have a negligible effect on current data. Also, with a PMHS model having a more close resemblance to the in vivo situation, current findings could present important data to support future choices in clinical practice.

## Conclusion

The ICP recordings of the four terminal devices were comparable, when tested with a standardized pressurization model in a PMHS model. None of the included terminal devices or needle types were found to be superior. The results provide evidence for more diverse material selection when logistic choices for ICP measurement devices are warranted.

## Supplementary Information

**Additional file 1: Supplementary Table 1.** Different needle types used in the experimental setup.

**Additional file 2: Supplementary Figure 1.** Graphical illustration of the pressure recordings for the four terminal devices (Compass manometer, the Stryker device, Meritrans transducer, and an arterial line) in combination with a Styker side-ported needle (A), C2Dx slit catheter (B), and a Sonoplex straight needle (21 gauge; C).

## Data Availability

The datasets used and/or analysed during the current study are available from the corresponding author on reasonable request.

## Abbreviations

CECS: Chronic exertional compartment syndrome
ICP: Intracompartmental pressure
PMHS: Post-mortem human specimen
