# Supplementary material for: Applicability of devices available for the measurement of intracompartmental pressures: a cadaver study
Source: J Exp Orthop. 2022 Sep 27;9:98. doi: 10.1186/s40634-022-00529-0 (PMC9515326; doi:10.1186/s40634-022-00529-0)
Supplement: Supplementary file 1 — Additional file 1: Supplementary Table 1. Different needle types used in the experimental setup. [file 40634_2022_529_MOESM1_ESM.docx]

**SUPPLEMENTARY TABLE 1 –** Different needle types used in the experimental setup.

|  | Company | Tip | Diameter (in Gauge) | Length (in mm) | Intentional use |
| --- | --- | --- | --- | --- | --- |
| Catheters |  |  |  |  |  |
| 1. Slit Catheter | C2Dx | Axially cut slits | 18 | 305 | Intracompartmental pressure measurement |
| 1. Venflon | Becton, Dickinson and Company | Blunt | 22 | 25 | Venous catheter |
| 1. Venflon | Becton, Dickinson and Company | Blunt | 20 | 32 | Venous catheter |
| 1. Venflon | Becton, Dickinson and Company | Blunt | 18 | 32 | Venous catheter |
| 1. Venflon | Becton, Dickinson and Company | Blunt | 17 | 45 | Venous catheter |
| 1. Venflon | Becton, Dickinson and Company | Blunt | 16 | 45 | Venous catheter |
| 1. Venflon | Becton, Dickinson and Company | Blunt | 14 | 45 | Venous catheter |
| 1. Intranule | Vygon | Blunt | 18 | 105 | Venous catheter |
| 1. Intranule | Vygon | Blunt | 16 | 105 | Venous catheter |
| 1. Intranule | Vygon | Blunt | 14 | 105 | Venous catheter |
| 1. Intranule | Vygon | Blunt | 13 | 105 | Venous catheter |
| Straight needles |  |  |  |  |  |
| 1. Microlance | Becton, Dickinson and Company | Bevel | 25 | 25 | Injection needle |
| 1. Microlance | Becton, Dickinson and Company | Bevel | 23 | 25 | Injection needle |
| 1. Mircolance | Becton, Dickinson and Company | Bevel | 21 | 50 | Injection needle |
| 1. Microlance | Becton, Dickinson and Company | Bevel | 18 | 40 | Injection needle |
| 1. Microlance | Becton, Dickinson and Company | Bevel | 16 | 40 | Injection needle |
| 1. Sonoplex | Pajunk | Facet | 22 | 100 | Nerve block application |
| 1. Sonoplex | Pajunk | Facet | 21 | 100 | Nerve block application |
| 1. Sonoplex | Pajunk | Facet | 20 | 150 | Nerve block application |
| Side-port needles |  |  |  |  |  |
| 1. Side-port needle | C2Dx | Bevel + side-port | 18 | 61 | Intracompartmental pressure measurement |
| 1. Sonoplex | Pajunk | Sprotte | 22 | 90 | Nerve block application |
| 1. Side-port needle | Stryker | Bevel + side-port | 18 | 61 | Intracompartmental pressure measurement |
