# Supplementary figures and images for: Applicability of devices available for the measurement of intracompartmental pressures: a cadaver study
Source: J Exp Orthop. 2022 Sep 27;9:98. doi: 10.1186/s40634-022-00529-0 (PMC9515326; doi:10.1186/s40634-022-00529-0)

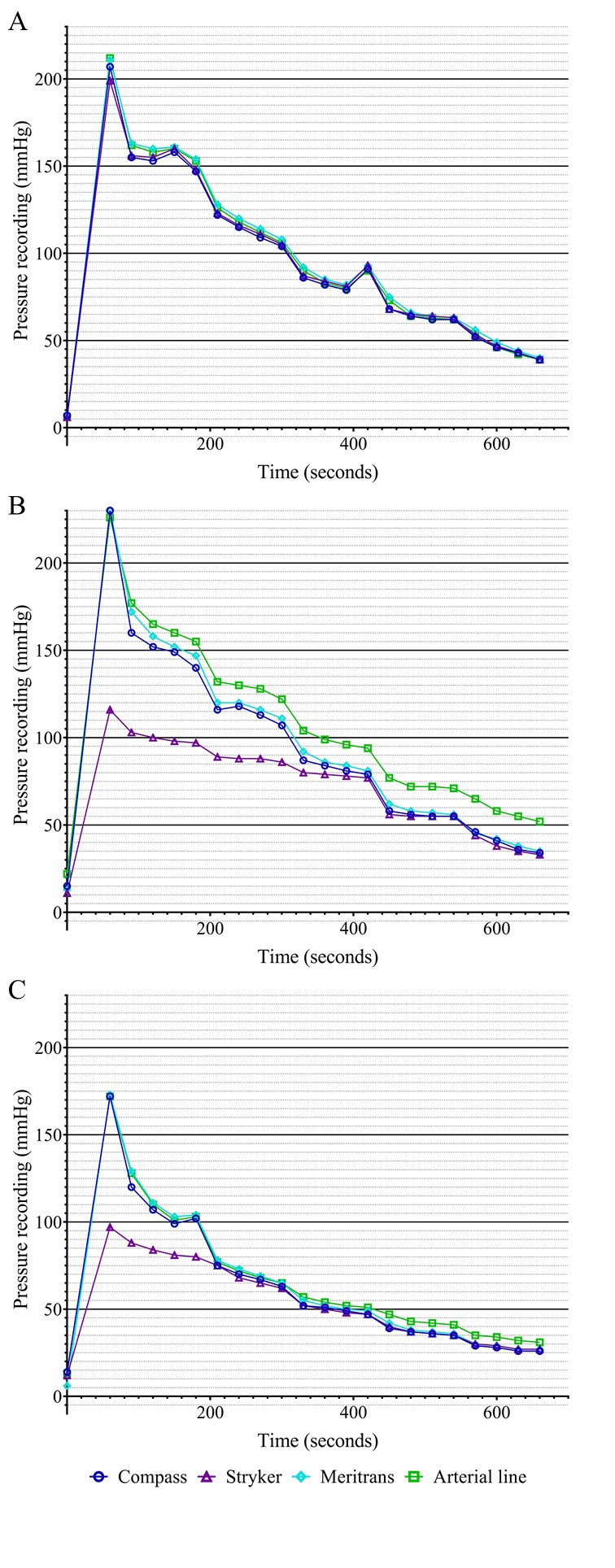

Supplement: Supplementary file 2 — Additional file 2: Supplementary Figure 1. Graphical illustration of the pressure recordings for the four terminal devices (Compass manometer, the Stryker device, Meritrans transducer, and an arterial line) in combination with a Styker side-ported needle (A), C2Dx slit catheter (B), and a Sonoplex straight needle (21 gauge; C). [file 40634_2022_529_MOESM2_ESM.jpg]
